# A Meta-Analysis of the Association between* DNMT1* Polymorphisms and Cancer Risk

**DOI:** 10.1155/2017/3971259

**Published:** 2017-04-03

**Authors:** Hao Li, Jing-wei Liu, Li-ping Sun, Yuan Yuan

**Affiliations:** Tumor Etiology and Screening Department, Cancer Institute and General Surgery, The First Affiliated Hospital of China Medical University, Key Laboratory of Cancer Etiology and Prevention (China Medical University), Liaoning Provincial Education Department, Shenyang, Liaoning 110001, China

## Abstract

Previous studies have examined the associations of DNA methyltransferase 1 (*DNMT1*) polymorphisms, including single nucleotide polymorphisms rs16999593 (T/C), rs2228611 (G/A), and rs2228612 (A/G), with cancer risk. However, the results are inconclusive. The aim of this meta-analysis is to elucidate the associations between* DNMT1* polymorphisms and cancer susceptibility. The PubMed, Embase, Web of Science, and Chinese National Knowledge Infrastructure databases were searched systematically to identify potentially eligible reports. Odd ratios and 95% confidence intervals were used to evaluate the strength of association between three* DNMT1* polymorphisms and cancer risk. A total of 16 studies were finally included in the meta-analysis, namely, nine studies of 3378 cases and 4244 controls for rs16999593, 11 studies of 3643 cases and 3866 controls for rs2228611, and three studies of 1343 cases and 1309 controls for rs2228612. The* DNMT1* rs2228612 (A/G) polymorphism was significantly related to cancer risk in the recessive model. The meta-analysis also suggested that* DNMT1* rs16999593 (T/C) may be associated with gastric cancer, while rs2228611 (G/A) may be associated with breast cancer. In future research, large-scale and well-designed studies are required to verify these findings.

## 1. Introduction

DNA methylation is one of the most commonly occurring epigenetic events in the mammalian genome. DNA methyltransferases (*DNMTs*) are critical to establishing and maintaining DNA methylation patterns by converting cytosine residues to 5-methylcytosine (5mC) in cytosine–guanine (CpG) dinucleotides [1, 2]. The DNMT family comprises three active forms: DNMT1, DNMT3A, and DNMT3B. DNMT1 is the major enzyme responsible for methylation maintenance [3, 4], while DNMT3A and DNMT3B are thought to function in de novo methylation rather than in methylation maintenance [3, 4].

*DNMT1* is located on human chromosome 19p13.2 and encodes a protein comprising 1632 amino acids, which may be implicated in occurrence progression and prognosis of the cancer. DNMT1 consists of three major structural elements: an N-terminal regulatory domain that is necessary for the localization of DNMT1; a C-terminal catalytic domain that is involved in the binding of substrates; and a central linker that contains repeated glycine-lysine dipeptides [5]. Genetic variation caused by single nucleotide polymorphisms (SNPs) is the most common form of altered gene structure. The most commonly studied* DNMT1* SNPs are rs16999593 (T/C), rs2228611 (G/A), and rs2228612 (A/G), which are present in coding regions and, therefore, may influence* DNMT1 *expression.

In recent years, various studies have indicated that* DNMT1* polymorphisms may play pivotal roles in carcinogenesis. The SNPs rs16999593 (T/C) and rs2228612 (G/A) were initially found to be associated with risk of breast cancer [6, 7], while SNP rs2228611 (A/G) was linked to gastric cancer [8]. Subsequently, a number of studies have concentrated on the relationships between* DNMT1* polymorphisms and risks of different cancers [6, 7, 9–21]. However, the results from these studies are inconsistent.

Until now, no meta-analysis has been carried out to investigate the relation of the three* DNMT1* polymorphisms (rs16999593 (T/C), rs2228611 (G/A), and rs2228612 (A/G)) with risk of cancer. Individual studies have lacked the ability to obtain overall reliable conclusions because of limited sample sizes and/or variations in ethnicities. To obtain further insights into the roles of* DNMT1* polymorphisms in carcinogenesis, we mainly performed a meta-analysis on the associations between these three SNPs and cancer risk.

## 2. Materials and Methods

### 2.1. Search Strategy

We systematically searched the PubMed, Embase, Web of Science, and Chinese National Knowledge Infrastructure databases using different combinations of the search terms “*DNMT1* or DNA methyltransferase 1,” “polymorphism or mutation or variant,” and “cancer or neoplasm or tumor.” The search was last updated on 06 June 2016. When overlapping data were found, only the largest and latest study was selected. We contacted the authors and requested their specific raw data when the data provided in the published article were not sufficient.

### 2.2. Inclusion and Exclusion Criteria

Studies were included when they met the following criteria: case-control study; the subject was the association of* DNMT1* polymorphisms (rs16999593 (T/C), rs2228611 (G/A), and rs2228612 (A/G)) with risk of cancer; and essential information on genotype or allele frequencies was available to assess the odds ratios (ORs) and 95% confidence intervals (CIs). Exclusion criteria included review articles; systematic reviews and meta-analyses; animal studies; sample size less than 100; and scarce or insufficient information on genotype or allele frequencies for the rs16999593 (T/C), rs2228611 (G/A), or rs2228612 (A/G) polymorphisms of* DNMT1* despite us contacting the authors.

### 2.3. Data Extraction

Two of the authors (HL and JL) independently selected the articles and extracted the original data using a standardized and consistent method. The following information was collected from each study: first author, year of publication, ethnicity of the subjects, cancer type, numbers of cases and controls, and genotyping methods. Conflicts were resolved after discussion and consensus was finally reached on all the extracted information.

### 2.4. Statistical Analysis

All statistical analyses were conducted using STATA software (version 12.0; Stata Corp LP, College Station, TX). ORs and their corresponding 95% CIs were employed to assess the strength of relationships between the* DNMT1* polymorphisms and cancer risk. *P* values < 0.05 were considered as statistically significant. Heterogeneity was calculated using the *Q* statistic (*P* value < 0.10 indicates significant heterogeneity among studies) and *I*-squared value. The Mantel-Haenszel fixed-effects model was used to calculate the pooled ORs when the heterogeneity of studies was not significant. Otherwise, the DerSimonian and Laird random-effects model was used. We conducted the sensitivity analysis to explore heterogeneity when significant heterogeneity existed. Subgroup analysis was applied to explore the effects of cancer type and genotyping method. In addition, Begg's test and Egger's test were performed to evaluate publication bias; *P* values < 0.05 for Begg's and Egger's tests indicate significant publication bias.

## 3. Results

### 3.1. Characteristics of the Studies

**Figure 1:**
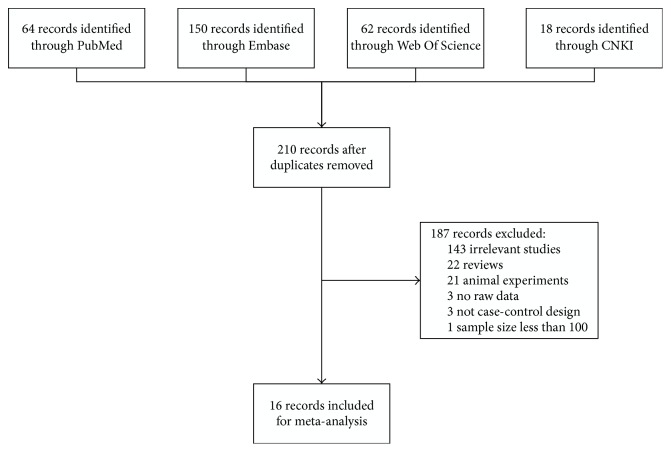
The flowchart of literature inclusion and exclusion.

This meta-analysis was organized according to the PRISMA (Supplementary File 1 in Supplementary Material available online at https://doi.org/10.1155/2017/3971259). A detailed flow chart of the study selection process is shown in Figure 1. A total of 215 potentially relevant articles were found by searching the four databases and after removing duplicates. Altogether 187 publications were excluded mainly due to no relevance, animal not human experiments, reviews, or meeting abstract. The 28 remaining articles were evaluated further for eligibility. Finally, 16 articles were included in the present meta-analysis [6–21].

The baseline characteristics of the 16 included studies are summarized in Table 1. Among them, nine articles containing 3378 cases and 4244 controls surveyed the association between rs16999593 (T/C) and cancer risk [6, 7, 9–15]; 11 studies on the relation between rs2228611 (G/A) and cancer risk included 3643 cases and 3866 controls [6–8, 10, 11, 13, 14, 18–21]; and three publications containing 1343 cases and 1309 controls explored the correlation of rs2228612 (A/G) with cancer risk [7, 16, 17]. The populations surveyed in the nine rs16999593 (T/C) studies were all Chinese [6, 7, 9–15]. In the rs2228611 (G/A) studies, nine of the populations were Chinese [6, 7, 10, 11, 13, 14, 19–21], one was Iranian [8], and one was Polish [18]. In the rs2228612 (A/G) studies the populations were either Caucasian [16] or Chinese [7, 17]. The genotyping methods used to detect the* DNMT1* polymorphisms included sequencing, MassARRAY, PCR-RFLP, MALDI-TOF, TaqMan, and SNPlex [6–21]. We used subgroup analyses to explore the effects of different cancer types and genotyping methods on the associations of increased risk of cancer with the* DNMT1* rs16999593 (T/C) and rs2228611 (G/A) polymorphisms. We did not perform subgroup analysis for rs2228612 (A/G) because of the limited number of articles that was available.

### 3.2. Quantitative Data Synthesis

**Figure 2:**
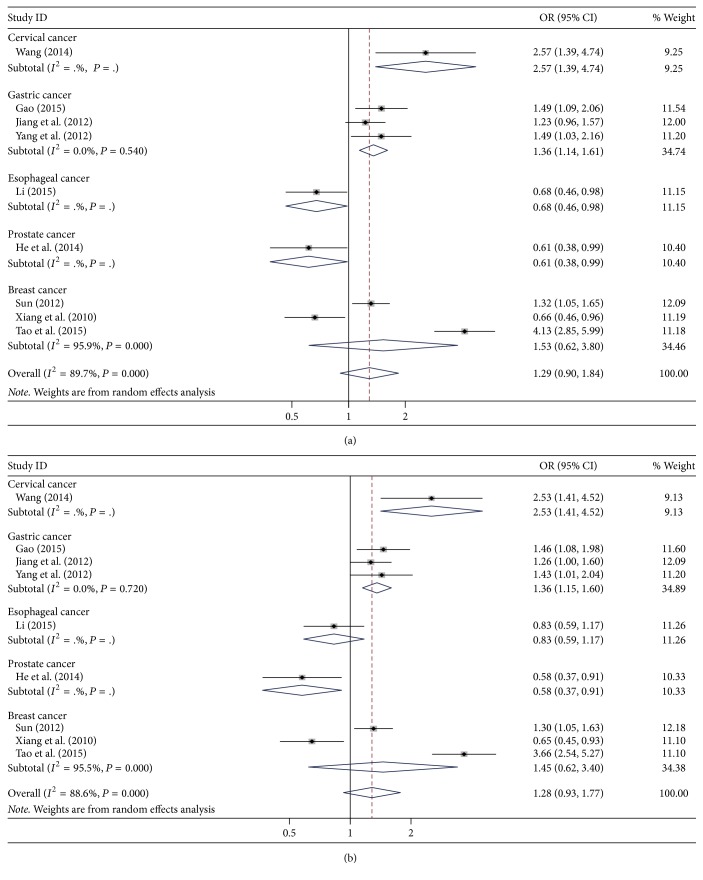
Forest plot for the association between* DNMT1* rs16999593 (T/C) polymorphism and cancer risk in the cancer type subgroup. (a) TC versus TT; (b) TC + CC versus TT.

The results for the association of* DNMT1* rs16999593 (T/C) with cancer risk are summarized in Table 2. Overall, neither the heterozygous nor dominant genetic models found significant associations between rs16999593 (T/C) and cancer risk (TC versus TT: OR = 1.29, 95% CI = 0.90–1.84, *P* = 0.163; TC + CC versus TT: OR = 1.28, 95% CI = 0.93–1.77, *P* = 0.135). The allele analysis also found no significant association (C allele versus T allele: OR = 1.18, 95% CI = 0.96–1.45, *P* = 0.127). For the subgroup analysis according to cancer type (Figure 2), rs16999593 (T/C) was consistently associated with increased risk of gastric cancer (TC versus TT: OR = 1.36, 95% CI = 1.14–1.61, *P* = 0.001; TC + CC versus TT: OR = 1.36, 95% CI = 1.15–1.60, *P* < 0.001; C allele versus T allele: OR = 1.28, 95% CI = 1.11–1.47, *P* = 0.001), but no significant association was found with breast cancer. For the different genotyping methods, rs16999593 (T/C) demonstrated increased risk of cancer in the sequencing subgroup but not in the MassARRAY subgroup.

**Figure 3:**
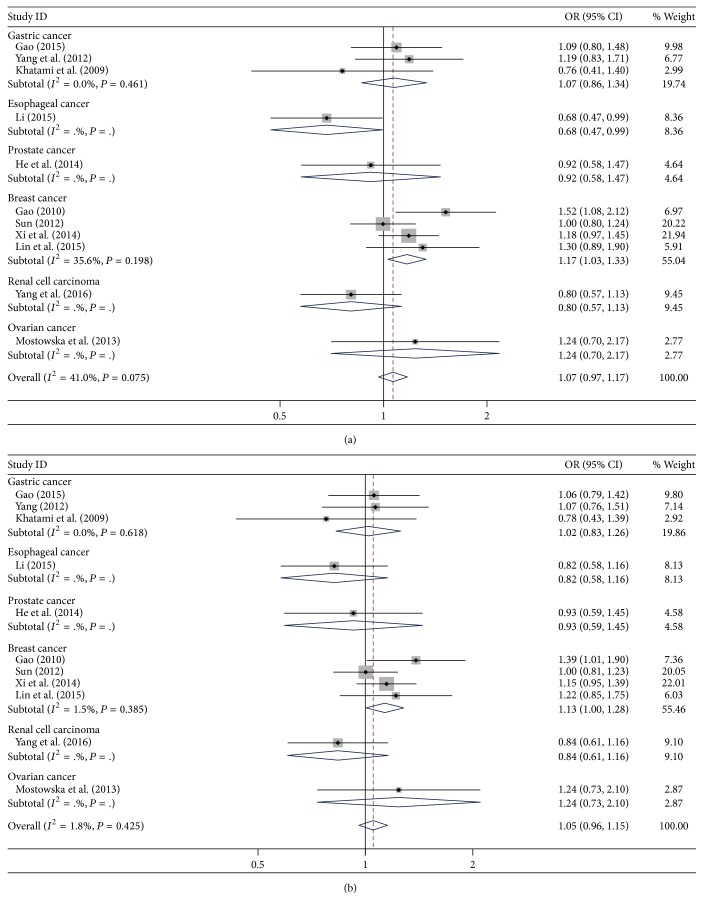
Forest plot for the association between* DNMT1* rs2228611 (G/A) polymorphism and risk of cancer in the subgroup of cancer type. (a) GA versus GG; (b) GA + AA versus GG.

The results for the association of* DNMT1* rs2228611 (G/A) with cancer risk are summarized in Table 3. Overall, the GA genotype was not significantly associated with risk of cancer compared with the GG genotype (OR = 1.05, 95% CI = 0.92–1.21, *P* = 0.075), and the GA + AA genotype was not related to cancer risk compared with the GG genotype (OR = 1.05, 95% CI = 0.96–1.15, *P* = 0.284). Similarly, no significant association was observed in the allele analysis (A allele versus G allele: OR = 1.02, 95% CI = 0.95–1.10, *P* = 0.532). In the subgroup analysis according to cancer type (Figure 3), the rs2228611 (G/A) was associated with higher risk of breast cancer (GA versus GG: OR = 1.17, 95% CI = 1.03–1.33, *P* = 0.016; GA + AA versus GG: OR = 1.13, 95% CI = 1.00–1.28, *P* = 0.043). A similar result was found in the MALDI-TOF subgroup (GA versus GG: OR = 1.20, 95% CI = 1.03–1.41, *P* = 0.022) in the analysis according to different genotyping methods. For the gastric cancer and PCR-RFLP subgroups, no significant associations were found in any of the compared genetic models.

**Figure 4:**
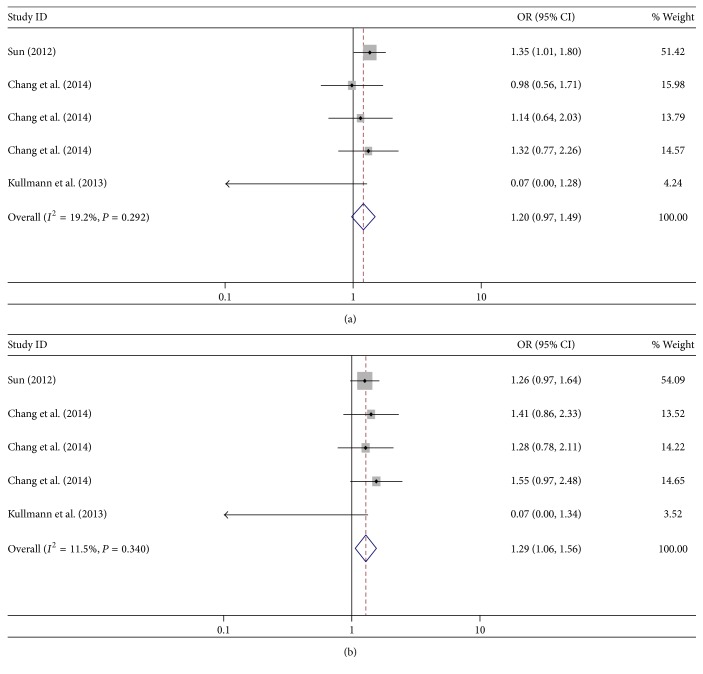
Forest plot for the association between* DNMT1* rs2228612 (A/G) polymorphism and cancer risk. (a) GG versus AA; (b) GG versus AA + AG.

The results for the association of* DNMT1* rs2228612 (A/G) with cancer risk are summarized in Table 4 and Figure 4. Significant association with cancer risk was observed in the recessive model (GG versus AG + AA: OR = 1.29, 95% CI = 1.06–1.56, *P* = 0.009), but no significant association was revealed in the allele analysis (G allele versus A allele: OR = 1.00, 95% CI = 0.83–1.20, *P* = 0.980).

### 3.3. Heterogeneity Test and Sensitivity Analysis

In most of the comparisons of DNMT1 rs16999593 and rs2228612 polymorphisms and one comparison of DNMT1 rs2228611, significant heterogeneity was observed. We next performed a leave-one-out sensitivity analysis. The results show that no individual study significantly affected the pooled OR (figure not shown), suggesting that the results of the meta-analysis were robust.

### 3.4. Publication Bias

Begg's test and Egger's test were used to quantitatively evaluate the publication bias of the selected studies; the details are listed in Table 5. For the associations of* DNMT1* rs16999593 (T/C), rs2228611 (G/A), and rs2228612 (A/G) with cancer risk, rs2228612 (A/G) showed publication bias (GG versus AA; G allele versus A allele) and rs16999593 (T/C) showed publication bias in the comparison with the recessive model.

## 4. Discussion

DNMT1, the major methyltransferase in mammals, lies in the replication fork and methylates newly synthesized DNA strands directly in S phase of DNA replication sites [22], which is essential for epigenetic inheritance. Regional aberrant DNA hypermethylation has been identified as a possible mechanism of inactivation of tumor suppressor genes [23]. Many studies have indicated that the overexpression of* DNMT1* could silence vital tumor suppressor genes such as* APC*,* P16*, and* RUNX3* through DNA methylation [24, 25]. Therefore, DNMT1 might be implicated in the occurrence, development, and prognosis of multiple types of cancer.

Polymorphisms have been identified as a powerful tool for predicting hereditary susceptibility of some complex diseases including cancer. However, previous individual studies about the association between* DNMT1* polymorphisms and cancer risk were not only limited but also inconclusive. To our knowledge, this is the first comprehensive meta-analysis investigating the possible correlations of SNPs rs16999593 (T/C), rs2228611 (G/A), and rs2228612 (A/G) in* DNMT1* with risk of overall cancer and specific cancer types, which is anticipated to shed light on the role of* DNMT1* polymorphisms in carcinogenesis.

SNP rs2228612 (A/G) causes an isoleucine to phenylalanine substitution at amino acid 327 in the DNMT1 protein, which may alter the function of DNMT1 and influence its effect in the carcinogenesis. In this meta-analysis, we found that* DNMT1* rs2228612 (A/G) was associated with risk of overall cancer in the recessive model. However, only three articles with small-scale studies were available for analysis; therefore, the results should to be interpreted with caution.

We did not find significant association between SNP rs16999593 (T/C) and overall cancer risk in any genetic comparison. Different types of cancer have distinct initiation and progression mechanisms, in which polymorphisms in key genes play critical roles. This meta-analysis elucidated that the* DNMT1* rs16999593 (T/C) polymorphism was associated with different cancer types. In the subgroup analysis according to cancer type, the TC genotype of rs16999593 (T/C) was associated with risk of gastric cancer, but not breast cancer. Extensive evidence has suggested that DNA methylation is involved in the initiation and progression of gastric cancer and increased expression of* DNMT1* had been confirmed to be related to gastric cancer [25]. The AKT-NF*κ*B and STAT3 signaling pathways can upregulate* DNMT1* expression, which could cause aberrant DNA methylation of tumor suppressor genes and lead to gastric cancer [26, 27]. Therefore, SNP rs16999593 (T/C), which causes a histidine to arginine substitution at 97 positions of the amino acid sequence, might affect the function of DNMT1, thus increasing susceptibility to gastric cancer. The subgroup of breast cancer involved two types of breast cancer: sporadic triple-negative breast cancer (TNBC) [15] and infiltrating ductal beast carcinoma (IDBC) [6]. The TC genotype of rs16999593 (T/C) was related to increased TNBC risk but decreased IDBC risk. Therefore,* DNMT1* might have diverse functions in different types of breast cancer. Further studies of the effects of* DNMT1* polymorphisms on specific breast cancer types are still needed. In addition, only in the subgroup of sequencing,* DNMT1* rs16999593 (T/C) were constantly associated with increased cancer risk in all compared genetic models, indicating that different genotype detecting methods might influence the results.

According to the functional prediction tool F-SNP (http://compbio.cs.queensu.ca/F-SNP/), SNP rs2228611 (G/A) may change the regulation of* DNMT1* splicing by leading to a synonymous mutation at amino acid 463. Multiple transcript variants of DNMT1 gene as a result of alternative splicing have been found. Therefore, we speculated that the rs2228611 (G/A) might influence the process of carcinogenesis by regulating the pattern of alternative splicing of* DNMT1*. Here, we did not find any significant associations between* DNMT1* rs2228611 (G/A) with altered risk of cancer in any genetic model. However, in the subgroup analysis, individuals with the GA genotype of rs2228611 (G/A) were associated with higher risk of breast cancer in both heterozygous and dominant models. In addition, individuals with the GA genotype of rs2228611 (G/A) had decreased risk in one study on esophageal cancer [14], which was opposite to the results for breast cancer. This reverse outcome may be because various types of cancer have different mechanisms of carcinogenesis. Future studies on different types of cancer may help to better understand these findings.

Some limitations of our meta-analysis should be noted. Firstly, the number of studies was not sufficiently large, especially for subgroup analysis of* DNMT1* rs2228612 (A/G). Secondly, the languages of the publications were limited to English and Chinese. Thirdly, although this meta-analysis was based on the whole population, most studies were from Chinese populations, except for one Iranian case and one Polish case for rs2228611 (G/A) and another one Caucasian case for rs2228612 (A/G), which showed the same results with the Chinese population. So this study had a certain universality, especially for Chinese population. Finally, publication bias was found in two comparisons of rs2228612 (A/G) and one comparison of rs16999593 (T/C).

## 5. Conclusion

The* DNMT1* rs2228612 (A/G) GG genotype may be associated with increased risk of cancer compared with the AA + AG genotype. SNP rs16999593 (T/C) is significantly associated with gastric cancer risk while SNP rs2228611 (G/A) is significantly related to breast cancer risk. Further large-scale and well-designed investigations including different cancers are required to verify the findings of this study.

## Supplementary Material

PRISMA is an evidence-based minimum set of items for reporting in systematic reviews and meta-analyses.

## Figures and Tables

**Table 1:** Characteristics of the included studies in this meta-analysis.

Author	Year	Ethnicity	Cancer type	Genotyping method	Case				Control			
					Total	MM	WM	WW	Total	MM	WM	WW
*For DNMT1 rs16999593 T/C polymorphism*												
Wang [12]	2014	Chinese	Cervical cancer	Sequencing	100	48	44	8	100	70	25	5
Gao [13]	2015	Chinese	Gastric cancer	Sequencing	310	180	112	18	420	281	117	22
Li [14]	2015	Chinese	Esophageal cancer	MassARRAY	258	138	80	40	260	127	109	24
He et al. [11]	2014	Chinese	Prostate cancer	MassARRAY	155	94	53	8	155	73	67	15
Sun [7]	2012	Chinese	Breast cancer	MassARRAY	1327	425	224	29	1440	504	202	28
Xiang et al. [6]	2010	Chinese	Breast cancer	PCR-RFLP	305	239	64	2	314	220	89	5
Jiang et al. [9]	2012	Chinese	Gastric cancer	Sequencing	447	283	144	20	961	659	273	29
Yang et al. [10]	2012	Chinese	Gastric cancer	MALDI-TOF	242	141	89	12	294	196	83	15
Tao et al. [15]	2015	Chinese	Breast cancer	Sequencing	234	68	164	2	300	180	105	15
*For DNMT1 rs2228611 G/A polymorphism*												
Gao [13]	2015	Chinese	Gastric cancer	Sequencing	310	167	128	15	420	232	163	25
Li [14]	2015	Chinese	Esophageal cancer	MassARRAY	258	131	85	42	260	119	113	28
He et al. [11]	2014	Chinese	Prostate cancer	MassARRAY	155	82	61	12	155	79	64	12
Xiang et al. [6]	2010	Chinese	Breast cancer	PCR–RFLP	305	125	149	31	314	154	121	39
Yang et al. [21]	2016	Chinese	Renal cell carcinoma	PCR–RFLP	293	152	117	24	293	139	133	21
Yang et al. [10]	2012	Chinese	Gastric cancer	MALDI-TOF	242	132	97	13	285	160	99	26
Sun [7]	2012	Chinese	Breast cancer	MassARRAY	678	341	279	58	733	369	303	61
Mostowska et al. [18]	2013	Polish	Ovarian cancer	PCR–RFLP	159	28	74	57	210	44	94	72
Xi et al. [19]	2014	Chinese	Breast cancer	MALDI-TOF	810	385	362	63	848	432	343	73
Lin et al. [20]	2015	Chinese	Breast cancer	MALDI-TOF	233	107	109	17	236	120	94	22
Khatami et al. [8]	2009	Iranian	Gastric cancer	PCR–RFLP	200	34	50	16	112	32	62	18
*For DNMT1 rs2228612 A/G polymorphism*												
Sun [7]	2012	Chinese	Breast cancer	MassARRAY	675	254	273	148	731	308	290	133
Chang et al. [17]	2014	Chinese	Esophageal cancer	SNPlex	137	52	56	29	357	100	200	57
Chang et al. [17]	2014	Chinese	Stomach cancer	SNPlex	143	43	72	28	357	100	200	57
Chang et al. [17]	2014	Chinese	Liver cancer	SNPlex	158	48	74	36	357	100	200	57
Kullmann et al. [16]	2013	Caucasian	Breast cancer	TaqMan	221	193	28	0	221	180	35	6

Abbreviations: W, wild-type allele; M, mutant-type allele.

**Table 2:** Meta-analysis results of the association between *DNMT1* rs16999593 (T/C) polymorphism and cancer risk.

Genetic model	Group/subgroup	*N*	Heterogeneity test		Statistical model	Test for overall effect	
			*I*_2_ (%)	*P*_het_		OR (95% CI)	*P*
CC versus TT	Overall	9	37.5	0.119	F	1.17 (0.92–1.49)	0.213
	Gastric cancer	3	0.0	0.743	F	1.36 (0.93–1.99)	0.117
	Breast cancer	3	48.1	0.146	F	0.93 (0.58–1.48)	0.748
	Sequencing	4	30.1	0.232	F	1.33 (0.90–1.95)	0.149
	MassARRAY	3	66.1	0.052	R	1.01 (0.53–1.93)	0.968

TC versus TT	Overall	9	89.70	<0.001	R	1.29 (0.90–1.84)	0.163
	Gastric cancer	3	00.00	0.540	F	**1.36 (1.14**–**1.61)**	**0.001**
	Breast cancer	3	95.90	<0.001	R	1.53 (0.62–3.80)	0.360
	Sequencing	4	90.30	<0.001	R	**2.06 (1.16**–**3.65)**	**0.013**
	MassARRAY	3	85.40	0.001	R	0.84 (0.49–1.43)	0.517

(TC + CC) versus TT	Overall	9	88.60	<0.001	R	1.28 (0.93–1.72)	0.135
	Gastric cancer	3	00.00	0.720	F	**1.36 (1.15**–**1.60)**	**<0.001**
	Breast cancer	3	95.50	<0.001	R	1.45 (0.62–3.40)	0.388
	Sequencing	4	88.40	<0.001	R	**1.99 (1.20**–**3.28)**	**0.007**
	MassARRAY	3	83.60	0.002	R	0.88 (0.55–1.41)	0.603

CC versus (TC + TT)	Overall	9	48.7	0.049	R	1.03 (0.72–1.49)	0.861
	Gastric cancer	3	0.00	0.635	F	1.22 (0.84–1.78)	0.303
	Breast cancer	3	70.5	0.034	R	0.49 (0.13–1.76)	0.274
	Sequencing	4	62.9	0.044	R	1.00 (0.49–2.04)	0.998
	MassARRAY	3	65.8	0.054	R	1.10 (0.59–2.05)	0.767

C allele versus T allele	Overall	9	81.40	<0.001	R	1.18 (0.95–1.45)	0.127
	Gastric cancer	3	00.00	0.936	F	**1.28 (1.11**–**1.47)**	**0.001**
	Breast cancer	3	91.50	<0.001	R	1.18 (0.71–1.97)	0.529
	Sequencing	4	66.30	0.030	R	**1.53 (1.26**–**1.95)**	**<0.001**
	MassARRAY	3	81.10	0.005	R	0.96 (0.67–1.36)	0.805

Abbreviations: R, random effect model; F, fixed effect model.

**Table 3:** Meta-analysis results of the association between *DNMT1* rs2228611 (G/A) polymorphism and cancer risk.

Genetic model	Group/subgroup	Studies	Heterogeneity test		Statistical model	Test for overall effect	
			*I*_2_ (%)	*P*_het_		OR (95% CI)	*P*
AA versus GG	Overall	11	0.00	0.925	F	0.99 (0.84–1.19)	0.898
	Gastric cancer	3	0.00	0.774	F	0.74 (0.49–1.13)	0.165
	Breast cancer	4	0.00	0.979	F	0.98 (0.78–1.22)	0.848
	PCR–RFLP	4	0.00	0.880	F	1.04 (0.76–1.42)	0.803
	MALDI-TOF	3	0.00	0.511	F	0.87 (0.65–1.17)	0.360

GA versus GG	Overall	11	41.00	0.075	R	1.05 (0.92–1.21)	0.445
	Gastric cancer	3	0.00	0.461	F	1.07 (0.86–1.33)	0.522
	Breast cancer	4	35.60	0.198	F	**1.17 (1.03**–**1.33)**	**0.016**
	PCR–RFLP	4	64.00	0.040	R	1.05 (0.73–1.52)	0.782
	MALDI-TOF	3	0.00	0.910	F	**1.20 (1.03**–**1.41)**	**0.022**

(GA + AA) versus GG	Overall	11	1.80	0.425	F	1.05 (0.96–1.15)	0.284
	Gastric cancer	3	0.00	0.618	F	1.02 (0.83–1.26)	0.860
	Breast cancer	4	1.50	0.385	F	**1.13 (1.00**–**1.28)**	**0.043**
	PCR–RFLP	4	51.70	0.102	F	1.06 (0.87–1.29)	0.542
	MALDI-TOF	3	0.00	0.872	F	1.14 (0.98–1.33)	0.087

AA versus (GA + GG)	Overall	11	0.00	0.596	F	0.97 (0.83–1.13)	0.671
	Gastric cancer	3	0.00	0.535	F	0.76 (0.51–1.12)	0.169
	Breast cancer	4	0.00	0.813	F	0.90 (0.73–1.12)	0.351
	PCR–RFLP	4	0.00	0.779	F	0.99 (0.76–1.29)	0.933
	MALDI-TOF	3	0.00	0.501	F	0.80 (0.60–1.06)	0.126

A allele versus G allele	Overall	11	0.00	0.978	F	1.02 (0.95–1.10)	0.532
	Gastric cancer	3	0.00	0.863	F	0.96 (0.82–1.14)	0.660
	Breast cancer	4	0.00	0.861	F	1.06 (0.96–1.16)	0.249
	PCR–RFLP	4	0.00	0.530	F	1.03 (0.90–1.18)	0.696
	MALDI-TOF	3	0.00	0.745	F	1.04 (0.93–1.17)	0.494

R: random effect model; F: fixed effect model.

**Table 4:** Meta-analysis results of the association between *DNMT1* rs2228612 (A/G) polymorphism and cancer risk.

Genetic model	Heterogeneity test		Statistical model	Test for overall effect	
	*I*_2_ (%)	*P*_het_		OR (95% CI)	*P*
GG versus AA	19.20	0.292	F	1.20 (0.97–1.49)	0.088
AG versus AA	60.30	0.039	R	0.81 (0.61–1.08)	0.156
(GG + AG) versus AA	62.7	0.030	R	0.87 (0.66–1.14)	0.310
GG versus (AA + AG)	11.50	0.340	F	**1.29 (1.06**–**1.56)**	**0.009**
G allele versus A allele	58.70	0.046	R	1.00 (0.83–1.20)	0.980

R: random effect model; F: fixed effect model.

**Table 5:** Results of publication bias test.

Polymorphism	Compared genotype	Begg's test		Egger's test	
		*z*-value	*P* value	*t*-value	*P* value
*DNMT1* rs16999593 T/C	CC versus TT	0.94	0.348	−2.00	0.085
	TC versus TT	0.10	0.917	0.03	0.976
	(TC + CC) versus TT	0.10	0.917	0.02	0.982
	CC versus (TC + TT)	1.98	0.048	−2.72	**0.030**
	C allele versus T allele	0.10	0.917	−0.45	0.669

*DNMT1* rs2228611 G/A	AA versus GG	1.40	0.161	−0.99	0.348
	GA versus GG	0.78	0.436	−0.65	0.530
	(GA + AA) versus GG	0.78	0.436	−0.69	0.505
	AA versus (GA + GG)	0.47	0.640	−0.61	0.556
	A allele versus G allele	0.93	0.350	1.17	0.273

*DNMT1* rs2228612 (A/G)	GG versus AA	1.71	0.086	−3.97	**0.029**
	AG versus AA	0.24	0.806	−2.86	0.065
	(GG + GA) versus AA	0.73	0.462	−3.81	**0.032**
	GG versus (AA + AG)	0.73	0.462	−1.37	0.263
	G allele versus A allele	2.20	**0.027**	−4.26	**0.024**
